# Launching an innovative educational model addressing substance use disorders and dental pain management (Project ECHO® in dentistry)

**DOI:** 10.1186/s12903-022-02417-6

**Published:** 2022-09-15

**Authors:** Richie Kohli, Karan Replogle, Andrea Gough-Goldman, Barry Taylor, Brandon Maughan, Harjit Singh Sehgal, Megan C. Herink, Rosemarie Hemmings, Sean Mahoney, Margaret McLain McDonnell, Kenneth McLemore, Eli Schwarz

**Affiliations:** 1 Department of Community Dentistry, School of Dentistry, Oregon Health & Science University, 3030 S. Moody Ave., Suite 135A, Portland, OR 97201-5042 USA; 2 Office of Clinical Affairs, School of Dentistry, Oregon Health & Science University, Portland, OR USA; 3 CODA Treatment Recovery (CODA Inc.), Portland, OR USA; 4 Oregon Dental Association, Wilsonville, OR USA; 5 Department of Emergency Medicine, School of Medicine, Oregon Health & Science University, Portland, OR USA; 6 Department of Periodontics, School of Dentistry, Oregon Health & Science University, Portland, OR USA; 7 College of Pharmacy, Oregon State University/Oregon Health & Science University, Portland, OR USA; 8 Mental Health and Addiction Association of Oregon, Portland, OR USA; 9 Oregon ECHO Network, Oregon Rural Practice-Based Research Network, Oregon Health & Science University, Portland, OR USA

**Keywords:** Interprofessional education, Case-based learning, Dental pain management, Opioids, Substance use disorder, Project ECHO model

## Abstract

**Background:**

Uninformed opioid prescribing by dentists has contributed to the current opioid crisis. This report describes the development and implementation of an innovative, interactive, multidisciplinary, and participant-centric telementoring program “Pain Management and Substance Use Disorders Dental ECHO (Extension for Community Health Care Outcomes)”. We disseminated information to dentists about appropriate opioid prescribing practices and engaged them with a focus on pain management and substance use disorders. The objective of this study was to assess the effectiveness of this program for self-reported: (1) change in knowledge and confidence related to clinical skills for dental pain management of patients with substance use disorders; (2) change in clinical behavior of dentists for safe opioids prescribing; and (3) change in clinic policies regarding safe opioids prescribing.

**Methods:**

An interdisciplinary panel of experts in medicine, pharmacy, social work, and dentistry designed and led the “Pain Management and Substance Use Disorders Dental ECHO” for invited dental care providers and dental students. Six cohorts each consisting of six, 1-h-long sessions were conducted via the Zoom videoconference platform in years 2020 and 2021. Each session included a didactic expert presentation, a participant-presented patient case and discussion. Each participant completed pre- and post-program surveys to assess the program’s influence on participant knowledge, clinical confidence and behavior change.

**Results:**

The participants (N = 151) were dentists (n = 109), dental faculty (n = 15), dental residents (n = 6), dental hygienists/assistants (n = 13) and nurses and clinic administrators (n = 8). Self-reported perceived medication knowledge, confidence in identification, treatment and willingness to engage with substance use disorders patients, and reported compliance with Prescription Drug Monitoring Program (PDMP) checks increased significantly from before to after the sessions (p < 0.001). Overall, participants expressed high levels of satisfaction with the content and reported that the sessions provided high benefit.

**Conclusion:**

The Project ECHO model is effective in rapidly disseminating evidence-based information. Dentists viewed this model as having a high degree of benefit for the optimal management of dental pain and the recognition and treatment of substance use disorders.

## Background

Opioid prescribing by dentists has contributed to the current opioid epidemic [1–3]. From 2010 to 2015, dentists prescribed 10–12% of all immediate-release opioids prescribed in the United States (U.S.) [1–3]. In 2016, the proportion of opioid prescriptions written by U.S. dentists was approximately 37 times higher than those written by English dentists [4]. Furthermore, dentists are the leading prescribers to young people, often coincident with the extraction of wisdom teeth [5, 6]. However, less than half of the opioids prescribed after surgical extractions are used, leaving opioids available for potential abuse or diversion [7–11]. Studies have also shown that US dentists have an exaggerated perception of the level of pain associated with dental procedures compared with what patients actually experience [12, 13], and almost a third of opioids prescribed are for non-surgical dental care [4]. Although opioid prescription rates in dentistry for Medicaid enrollees declined significantly between 2012 and 2019, the overall rate is still high and prescriptions are being written unnecessarily [14, 15]. Dental pain management in patients with substance use disorders is particularly challenging due to factors like multiple medical conditions and drug use, social history, concurrent chronic pain management by other providers, worries of drug misuse and diversion; and relapse during recovery [16]. Additionally, there remains a need for a more comprehensive and innovative approach to teaching and preparing dental students for treating patients with substance use and dependence in dental curriculums [17, 18].

In 2017, the US Department of Health and Human Services declared the opioid epidemic a national public health emergency [19]. Oregon, like the rest of the US, is experiencing an opioid crisis with more individuals reporting past year (2018–2019) opioid misuse (heroin and pain reliever misuse) than average US data (5.3 vs. 3.7%) [20]. For patient safety and effective pain management, Oregon Health Authority released clinical guidelines for dentists in 2016 on opioid prescribing [21]. Further, in Oregon, although it is a requirement that all Oregon licensed dentists who has an active Federal Drug Enforcement Administration (DEA) registration must register with the Prescription Drug Monitoring Program (PDMP), there is no requirement to check the PDMP before prescribing opioids [22]. To strengthen and further disseminate the existing efforts, the authors initiated an innovative collaborative dental continuing education project titled “Pain Management and Substance Use Disorders Dental ECHO (Extension for community health care outcomes)” co-sponsored by Oregon Health & Science University (OHSU) School of Dentistry and the Oregon Health Authority (OHA).

Project ECHO® is a well-established educational model for improving access to specialty care among underserved populations with complex conditions such as Hepatitis C [23], chronic pain, and HIV/AIDS) [24]. The medical community has successfully used ECHO nationally and globally to educate and share best practices with recent programming aimed at educating healthcare providers and raising awareness around safe, evidence-based pain management and substance use disorders [25, 26].

Project ECHO uses a simple videoconferencing platform to link community healthcare providers with disparate needs and levels of experience with a team of interdisciplinary experts for mutual learning [21]. The ECHO model allows for rapid dissemination of expert knowledge and best practices across distance using case-based learning.

The objective of this study was to assess the effectiveness of “Pain Management and Substance Use Disorders Dental ECHO” for self-reported:

1. Change in knowledge and confidence related to clinical skills for dental pain management of patients with substance use disorders;
2. Change in clinical behavior of dentists for safe opioids prescribing; and
3. Change in clinic policies regarding safe opioids prescribing.

## Methods

The project was reviewed and exempted by the Oregon Health & Science University Institutional Review Board (Study 00020289).

### Interdisciplinary team recruitment

**Fig 1:**
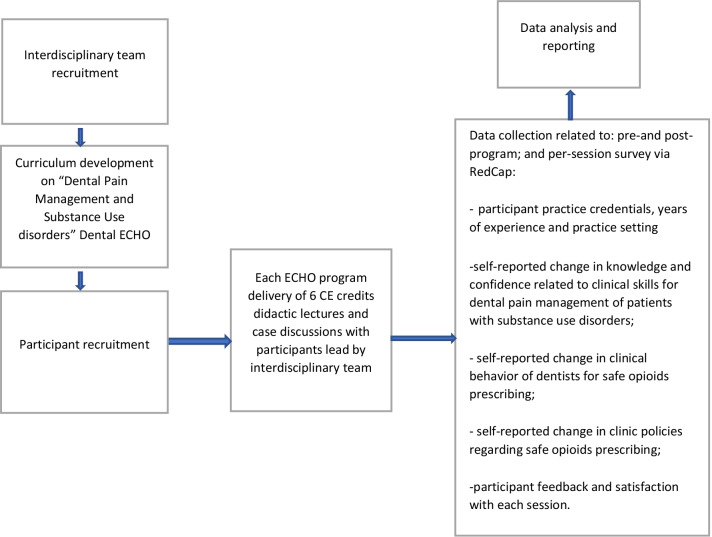
Study design

Program leaders recruited an interdisciplinary team of content experts from OHSU. (Fig. 1). The team included an emergency medicine physician, an addiction medicine physician, a clinical pharmacist, a social worker, a general dentist representing organized dentistry, and dental specialists (periodontist, endodontist, oral surgeon and dental public health provider). Each member of the team committed to adhering to the ECHO model and participation in the expert panel and presentation of a didactic session in their field of expertise.

### Curriculum development

The State Targeted Response (STR) Grant co-principal investigators (RK and KR) conducted an extensive review of the literature that was shared with the interdisciplinary team. The curriculum was finalized after a consensus meeting of the interdisciplinary team. The curriculum was designed as six, one-hour sessions with 10–15 min of a didactic presentation by a team member, followed by an interactive case discussion. The six session titles and objectives are described in Box 1. For case discussions, participants volunteered to present a patient scenario that illustrated the participants’ current challenge with safe prescribing, recognition of substance use disorders, or implementing harm reduction strategies. Patient cases were presented by participants from their clinical settings and experiences. Therefore, the topics included in each case varied from session to session based on what was presented by the participant and key questions identified. Some example topics discussed included: alternative medications to opioids in SUD patients, trauma informed care in SUD patients, treatment of acute pain in SUD patients, and communication strategies for difficult conversations with SUD patients.

**Box 1:** Didactic topics and objectives

Didactic topic	Objectives
Role of dentistry in combating opioid crisis/ Patient perceptions of pain and the need for opioids	To review the scope of the opioid epidemic as a national public health emergency
	To understand the role of dentistry and the interprofessional team in combating the opioid crisis
	To review and utilize best practices for effective and safe pain management
	To have a better understanding of pain and dentistry from a patient’s perspective
	To find more trauma-informed ways* to talk to patients with substance use disorders
	* Trauma-informed care ensures that the physical and emotional safety of an individual is addressed in the health care situation guided by choice, collaboration, trustworthiness, and empowerment.”
Identifying patients with substance use disorders	Identify behaviors of patients with substance use disorders
	Discuss prescription drug monitoring programs (PDMPs) and describe the use of the Oregon PDMP
	Describe the components of an SBIRT (Screening, Brief Intervention and Referral to Treatment) program
Pharmacological and non-pharmacological alternatives to opioids	Review evidence-based pharmacological and non-pharmacological alternatives to opioids for acute pain
	Discuss strategies for treating acute pain in patients on chronic opioids
	Describe options for the treatment of pain in opioid use disorders
Systems and protocols, diversion, disposal of medications	Describe dentists’ role in the compassionate and safe prescribing of opioids
	Identify racial and ethnic disparities related to the prescribing of analgesics and treatment for substance use disorders
	Discuss opioid diversion and safe disposal with patients and families
Harm reduction	Describe harm reduction
	Discuss how to apply harm reduction principles in an office visit
	Discuss how to reduce the harm that we as providers can cause
	Describe Naloxone—to whom it should be prescribed and how to prescribe it
	Provide a brief overview of the three medications for the treatment of opioid use disorders from a harm reduction perspective
Difficult conversations	Highlight communication strategies to approach difficult conversations with compassion
	Provide approaches to communicate opioid risks to patients more effectively and with less confrontation
	Identify interventions that can improve consistency in team prescription patterns and prevent future difficult conversations

The sessions and de-identified patient case presentations followed a standard format. The case was presented by the participant using a standard case presentation form. Other attending participants responded to the case by sharing verbally or in chat their experiences and recommendations for solutions to the case challenges. Following the discussion, the interdisciplinary team made expert recommendations, which were then formally posted on the participant ECHO resources virtual page. Each session was credentialed for one hour of free American Dental Association (ADA) Continuing Education Recognition Program (CERP) continuing education (CE) credit. The six sessions made-up one ECHO cohort, with the cohort participants meeting twice monthly.

### Participant recruitment and sample

OHSU faculty and residents (n = 176) received an invitation to participate via e-mail. The membership directory of the Oregon Dental Association (n = 630) and the Oregon Board of Dentistry’s list of licensed dentists (n = 279) with their e-mail addresses were used to invite dentists not directly associated with OHSU. Dentists were encouraged to participate with their staff members, including dental hygienists, administrators, and behavioral therapists. Dental providers in rural Oregon outside of Portland were also invited and encouraged to participate. A Web link was provided by the Oregon ECHO Network for registration.

Six cohorts of participants were recruited during the years 2020 and 2021 academic terms of Fall (Sep-Dec), Winter (Jan-Mar) and Spring (Apr-Jul).

### Measures

Each professional participant completed one pre- and one post-program survey soliciting information about participants’ professional and practice backgrounds. The following questions were contained in both the pre and the post survey:

- I am knowledgeable about the following medications: (Buprenorphine/ Combination of buprenorphine and naloxone/ Naloxone/ Naltrexone/ Acamprosate/ Disulfiram/ Methadone)
- Please choose the extent to which you disagree or agree with the following statement: I am confident in my skills to identify patients who may have a substance use disorder/ I am confident discussing pharmacological alternatives to opioids with patients/ I am confident discussing non-pharmacological alternatives to opioids with patients/ I am confident having difficult conversations with patients regarding their request for an opioid prescription
- Please choose the extent to which you disagree or agree with the following statement: I check the Prescription Drug Monitoring Program (PDMP) prior to prescribing opioids/ I serve as a local consultant within my clinic regarding alternatives to opioids
- Please choose the extent to which you disagree or agree with the following statement: My clinic has processes and procedures to administer the Screening, Brief Intervention, and Referral to Treatment (SBIRT) tool/ My clinic regularly promotes safer use practices (e.g. clean needles and supplies, naloxone) when appropriate.

The response options to the questions above were 1. Strongly Disagree, 2. Disagree, 3. Neutral (neither agree nor disagree), 4. Agree, 5. Strongly agree, 6. Not applicable (with non-applicable scores excluded from the calculations).

The following question was included to elicit respondents’ perceived confidence in their treatment of patients with different types of substance use disorder:

- I am [1. Not at all confident/ 2. Not very confident/ 3. Neutral (neither confident nor not confident)/ Moderately confident/ Extremely confident] treating patients with Opioid use disorder/ Alcohol use disorder/ Benzodiazepine disorder/ Methamphetamine use disorder/ Tobacco use disorder.

As seen, the response options were built into the survey question itself.

In addition, after each six sessions, participants were asked to assess the quality of the session content and didactic presentation, and their satisfaction with each session (per-session survey). Attendees addressed 6 aspects of the sessions: consistency between stated objectives and content, clear and concise presentation, presenters’ enthusiasm and knowledge; presenters’ willingness to take questions, each session’s ability to meet personal objectives and overall satisfaction. Response options were 1. Strongly Disagree, 2. Disagree, 3. Agree, 4. Strongly Agree.

All surveys were pilot-tested and refined prior to their implementation.

### Data analysis

All survey data were collected online via a RedCap® survey [27] and analyzed by SPSS®, version 25. Because the differences in practice credentials, years of experience, and practice setting between the cohorts were negligible, all cohorts were analyzed as one population. Data analysis included calculating pre- and post-mean scores for each Likert-item and using paired t-test of the mean scores to assess a statistically significant change in scores with values < 0.05 termed significant. In addition, the proportion of respondents who agreed or strongly agreed with a knowledge or behavior statement or declared they were moderately or extremely confident in treating patients with different types of substance use disorder was calculated and tabulated.

## Results

### Participant background

Six ECHO cohorts were conducted during the years 2020 and 2021. Altogether, 163 participants registered and attended the six cohorts. Out of those, 151 were included for analysis as they had completed pre-and post-program surveys with a mean attendance of 3.2 ± 1.7 sessions. An attrition issue of attendance was found in all the cohorts. Altogether for 6 cohorts, 120 attendees attended session 1 which gradually dropped to 78 attendees for session 6 (Table 1).

**Table 1:** Characteristics of professional participants in Dental ECHO program (across six cohorts)

Characteristic	Number	Percentage
*Credential*		
Dentist	109	72.1
FT Faculty	11	7.3
PT/Adj. Faculty	4	2.7
Dental Resident	6	4.0
Dental Hygienist/Dental Assistant	13	8.6
Other (Nurse, Admin)	8	5.3
Total	151	100.0
*Experience precepting students*		
Precepted in the past	52	34.4
Preceptor now	21	13.9
Anticipate being a preceptor	5	3.3
Not applicable	50	33.1
Missing information	23	15.2
Total	151	100.0
*Number of years practiced*		
1–5	16	10.5
6–10	32	21.2
11–15	34	22.51
16–20	34	22.5
20 +	15	9.9
Missing information	20	13.2
Total	151	100.0
*Practice setting*		
Federally Qualified Health Center (FQHC)	21	14.0
Federally Qualified Rural Health Center (FQRC)	20	13.2
Both FQHC and FQRC	28	18.5
Neither FQHC nor FQRC	56	37.1
Missing information	26	17.2
Total	151	100.0
*Participant attendance by session*		
Session 1	120	20.4
Session 2	116	19.7
Session 3	101	17.2
Session 4	91	15.5
Session 5	82	13.9
Session 6	78	13.3
151*6Total sessions = 906 (if all the participants attended all the sessions)	588	100.0

$$\overline{x }$$ = 3.23 ± 1.70  588/906 = 64.9%

Respondents who returned all pre- and post surveys = 89 (58.9%)

Respondents who returned some pre- or post surveys = 62 (41.1%)

Most participants were dental providers, including dentists (n = 109), dental faculty (n = 15), dental residents (n = 6) and dental hygienists/assistants (n = 13). Other participants were nurses and clinic administrators (n = 8) (Table 1). More than quarter of the participants (n = 41) participants worked at Federally Qualified Health Center (FQHC) and Federally Qualified Rural Health Center (FQRC). The number of years of practice experience for the professional participants ranged from 1 to 20 + . Close to half of the participants (n = 73) were either preceptors in the past or currently.

### Participants’ self-reported change in knowledge and confidence related to clinical skills for dental pain management of patients with substance use disorders

Participants reported increases in their perceived knowledge of all of the listed medications (buprenorphine, combination of buprenorphine and naloxone, naloxone, naltrexone, acamprosate, disulfiram, and methadone), Table 2. The pre-post changes varied in size with proportions ranging from 61.5 versus 81% for methadone and from 30.3 versus 71.3% for combination of buprenorphine and naloxone. All of the corresponding increases in mean scores in reported medication knowledge were statistically significant (p < 0.001).

**Table 2:** Professional participants’ change in knowledge of medications for substance use disorders and confidence in skills treating patients with substance use disorders before and after the ECHO program (overall average scores across six cohorts)

	N	Pre-program mean* (SD)	Percentage agree/strongly agree	Post-program mean* (SD)	Percentage agree/strongly agree	p value***
*Statement: I am knowledgeable about the following medications:*						
Buprenorphine	73	3.15 (1.11)	42.7	4.08 (0.83)	82.3	< 0.001
Combination of buprenorphine and naloxone	73	2.81 (1.05)	30.3	3.85 (0.95)	71.3	< 0.001
Naloxone	72	2.89 (1.10)	32.7	3.74 (1.08)	63.3	< 0.001
Naltrexone	73	2.16 (1.01)	7.3	3.07 (1.27)	38.8	0.002
Acamprosate	72	2.64 (1.14)	25.7	3.38 (1.09)	55.1	< 0.001
Disulfiram	83	3.07 (1.14)	41.6	3.96 (1.03)	76.3	< 0.001
Methadone	71	3.77 (1.21)	61.5	4.21 (0.89)	81.0	< 0.001
*Statement: Please choose the extent to which you disagree or agree with the following statement:*						
I am confident in my skills to identify patients who may have a substance use disorder	82	3.01 (1.12)	33.6	3.66 (1.08)	59.6	< 0.001
I am confident discussing pharmacological alternatives to opioids with patients	84	3.76 (0.87)	64.7	4.30 (0.66)	86.7	< 0.001
I am confident discussing non-pharmacological alternatives to opioids with patients	73	3.37 (1.23)	47.7	3.89 (1.04)	65.5	0.003
I am confident having difficult conversations with patients regarding their request for an opioid prescription	87	3.71 (0,99)	58.4	4.10 (0.88)	79.2	< 0.001
I am confident in my skills to identify patients who may have a substance use disorder	82	3.01 (1.12)	33.6	3.66 (1.08)	59.6	< 0.001

(1) Strongly disagree | (2) Disagree | (3) Neutral (neither agree nor disagree) | (4) Agree | (5) Strongly agree | (6) Not applicable *

^*^ Non-applicable scores were excluded from calculations

^***^Paired t-test, pre- and post-means (2-tailed)

Participants reported statistically significantly increased confidence in identifying patients who may have a substance use disorders (3.01 vs. 3.66, with the proportion agreeing or strongly agreeing with the statement increasing from 33.6 to 59.6%) (Table 2). Similarly, expressions of increased confidence were noted in relation to discussions of pharmacological and non-pharmacological alternatives to opioid prescriptions and difficult discussions with a patient concerning an opioid prescription (all mean scores increased significantly statistically, and considerable increases were found in participants agreeing or agreeing strongly with the statements) (Table 2).

Participants also reported that they felt considerably more confident in dental treatment of patients with different types of substance use disorders after completing the program (Table 3). Attendees reported higher levels of confidence when treating patients with methamphetamine use disorders with percentage reporting moderate/extreme confidence increasing from 31.5 to 68.2%, and mean scores increasing from 2.99 to 3.86 (p < 0.001). With regard to opioid use disorder, the percentage of attendees expressing moderate/extreme confidence increased from 38.7 to 72.8%, with mean scores increasing from 3.09 to 3.89 (p < 0.001). For benzodiazepine use disorders, the percentage of attendees expressing moderate/extreme confidence increased from 56.2 to 78.0%, with mean scores increasing from 3.46 to 4.14 (p < 0.001).

**Table 3:** Professional participants’ change in confidence in dental treatment of patients with different types of substance use disorders before and after the ECHO program (overall average scores across six cohorts)

Statement: I am _ treating patients with	N	Pre-program mean (SD)	Percentage moderately/extremely confident	Post-program mean (SD)	Percentage moderately/extremely confident	p value*
Opioid use disorder	88	3.09 (1.07)	38.7	3.89 (0.78)	72.8	< 0.001
Alcohol use disorder	89	3.16 (1.08)	40.0	3.77 (0.81)	68.0	< 0.001
Benzodiazepine use disorder	89	3.46 (1.02)	56.2	4.14 (0.76)	78.0	< 0.001
Methamphetamine use disorder	78	2.99 (1.05)	31.5	3.86 (0.86)	68.2	< 0.001
Tobacco use disorder	79	3.47 (1.06)	49.5	4.03 (0.91)	77.9	< 0.001

(1) Not at all confident | (2) Not very confident | (3) Neutral (neither confident nor not confident) | (4) Moderately confident | (5) Extremely confident

^*^Paired t-test, pre- and post-means (2-tailed):

### Participants’ self-reported change in clinical behavior for safe opioids prescribing

The participants reported significantly higher compliance with checking the PDMP prior to prescribing opioids (mean scores increased from 3.78 to 4.39, p < 0.001; proportion increased from 69.5 to 85.9%). On the other hand, there was negative post-program change with serving as local consultants within their clinics regarding alternatives to opioids (2.25 vs. 1.71, p = 0.52). (Table 4).

**Table 4:** Professional participants’ self-reported change in clinical behavior and system level changes in managing dental patients with substance use disorders before and after the ECHO program (overall average scores across six cohorts)

Statement: Please choose the extent to which you disagree or agree with the following statement:	N	Pre-program mean* (SD)	Percentage agree/strongly agree	Post-program mean* (SD)	Percentage agree/strongly agree	p value***
I check the Prescription Drug Monitoring Program (PDMP) prior to prescribing opioids	80	3.78 (1.10)	69.5	4.39 (0.82)	85.9	0.002
I serve as a local consultant within my clinic regarding alternatives to opioids	83	2.25 (1.33)	39.8	1.71 (1.03)	8.7	0.52
My clinic has processes and procedures to administer the Screening, Brief Intervention, and Referral to Treatment (SBIRT) tool	86	3.09 (1.18)	41.1	3.91 (1.10)	67.4	< 0.001
My clinic regularly promotes safer use practices (e.g. clean needles and supplies, naloxone) when appropriate	79	3.09 (1.08)	31.3	3.71 (1.10)	60.6	< 0.001

(1) Strongly disagree | (2) Disagree | (3) Neutral (neither agree nor disagree) | (4) Agree | (5) Strongly agree | (6) Not applicable

^*^Non-applicable scores were excluded from calculations

^***^Paired t-test, pre- and post-means (2-tailed)

### Participants’ self-reported change in clinic policies regarding safe opioids prescribing

Participants reported improved processes and procedures at their clinics to administer the Screening, Brief Intervention, and Referral to Treatment (SBIRT) tool (3.09 vs. 3.91, p < 0.001). The participants also reported statistically significant positive changes for clinics’ regular promotion of safer use practices (3.09 vs. 3.71, p < 0.001 (Table 4).

### Participants’ feedback

A total of 588 per-session surveys were completed by the participants regarding overall feedback on all sessions via per-session survey corresponding to a response rate of 64.9% (588/906) across all six cohorts. Overall, the participants expressed a high level of satisfaction with the program with mean scores above 3.4 on every session evaluation, and 96% or more of the respondents expressed agreement or strong agreement with the statements related to the quality of the session content and didactic presentations (Table 5). In open-ended responses, respondents highlighted that they were able to articulate practical learning in: (1) utilization of the PDMP ahead of prescribing, (2) caution when prescribing opioids, (3) modalities utilized for acute dental pain management (traditional and alternative), (4) communication skills to build trust and engage in difficult conversations with patients, (5) proper disposal of opioids and (6) ensuring naloxone is available in cases of overdose. Participants reported that knowledge gained in the program would impact their personal practice and prescribing behavior. They did not perceive their ability to impact more global factors, such as changing processes at a clinic or institutional level had improved. The participants also reported hesitation in active dissemination of new-found knowledge, such as holding in-clinic seminars and sharing information with colleagues.

**Table 5:** Professional participants’ perceptions of the contents and presenters in the ECHO sessions (overall average score across all sessions of six cohorts and all attendees)

Statement	Professional attendees (N = 147)	
	Mean (SD)	% Agree or strongly agree
Content was consistent with the publicized course objectives	3.54 (0.64)	97.3
Content was presented in a clear and concise manner	3.55 (0.64)	96.9
Presenter(s) was enthusiastic and knowledgeable about the subject	3.55 (0.64)	97.4
Presenter(s) encouraged questions and participation	3.57 (0.64)	98.2
My personal objectives for this session were met	3.48 (0.67)	95.8
Overall, I was satisfied with today's session	3.50 (0.66)	96.3

Likert scale, 1–4: 1, Strongly disagree; 2, Disagree; 3, Agree; 4, Strongly agree

A total of 588 per-session surveys by professional participants were analyzed as the per-session survey response was 64.9% (588/906) across all six cohorts

## Discussion

We aimed to initiate an innovative education program that could engage and involve practicing dentists to become more aware about opioid prescribing and its effects. Innovative, timely, easily accessed, and highly interactive training methodologies may empower behavior change by increasing providers’ knowledge and comfort level [28]. This Pain Management and Substance Use Disorders Dental ECHO demonstrated its potential to engage dental care providers to achieve a higher level of awareness, knowledge and self-efficacy in dealing with substance use disorders and pain management. Overall, as illustrated in the tables, participants reported increased knowledge, confidence, and expertise after completion of the program compared to before participation.

National, professional, and state clinical practice guidelines and legislation [21–24, 29–32] have been quite clear that the scientific evidence supports the use of over-the-counter analgesics such as ibuprofen and acetaminophen as first-line therapy for acute pain management in dentistry [33, 34]. In spite of expectations that prescribers will follow these recommendations more closely these steps are likely inadequate, as the overall rate of opioid prescriptions by dentists is still too high, especially in adolescents [6]. Further, as substance use disorders treatment options increase, dentists can play a significant role as an informed member of an interprofessional team of healthcare providers working to support individuals with a use disorder on their journey to recovery. In an earlier paper we identified possible barriers for dentists to take on this role, which might be linked to how the dental curriculum approach these topics [35]. The major barriers reported were that both dentists and dental students expressed reservations related to medication knowledge, ability to identify patients with substance use disorders and confidence in discussing substance use disorders with patients [36].

Thus, the challenge remains to find optimal methodologies for improving dental professionals’ knowledge, self-efficacy, and clinical behavior for the management of dental pain and for disseminating evidence-based best practices [37]. Our findings confirmed results of other Project ECHO studies on pain management. A recent study found that primary care providers who regularly attended Specialty Care Access Network-Extension for Community Health Outcomes pain management (SCAN-ECHO) sessions reported positive outcomes in terms of their confidence and knowledge in treating patients with chronic pain [28]. SCAN-ECHO was reported to potentially influence opioid-prescribing practices. A one-year trial in a large, multisite FQRHCs found that attendance at weekly Project ECHO pain sessions not only improved provider knowledge and self-efficacy, but also altered prescribing and referral patterns, suggesting that knowledge acquired during Project ECHO sessions translated into practice changes [36]. Finally, two systematic reviews analyzing a larger number of Project ECHO evaluation studies concluded that it is an effective and potentially cost-saving model that increases participant knowledge and patient access to health care in remote locations [38, 39]. Both reviews noted that further research examining the efficacy of the ECHO model is needed and that this need follows patterns similar to those of other service delivery models reported in the literature. Further, provider-related outcomes suggested favorable results across three domains: satisfaction, increased knowledge and increased clinical confidence [40].

With the drug addiction related overdose deaths continuing unabated [41], it is of high priority to ensure that the dental profession has access to and will utilize systems that can assist them to prevent unnecessary opioid prescribing. There is general agreement that PDMPs are effective in improving medical care; reducing doctor shopping, inappropriate prescribing, drug diversion and prescription fraud; and assisting in drug investigations [40, 42]. Such outcomes can contribute to lowering rates of addiction, overdose and deaths associated with misuse of prescription drugs, thus reducing the health care and public safety costs attributable to such misuse [43]. The reported increase in the use of the PDMP by these respondents (Table 4) indicate that it is feasible to convince dental professionals by appropriate practical examples to make use of this important state-based system in their clinical work.

Overall, participants reported benefiting from their participation in this Project ECHO and found the model satisfactory. They collectively agreed/strongly agreed that the content was consistent with the course objectives and was presented clearly, that the presenters were enthusiastic and encouraged participation, that their personal objectives were met and that they were satisfied with the sessions. The participants, however, did not report feeling empowered to serve as a local consultant within their clinic regarding alternatives to opioids. This may be because of the new information they learned throughout the course. Providing peer consultancy and advocating evidence-based and individualized patient-tailored approach in managing dental pain is important to bring effective and sustainable change at the profession and population level. Possibly, the cohorts were too brief to provide sufficient confidence in advocacy and institutional change. The successful implementation of the six ECHO cohorts focused mostly in Oregon generated increased interest from other states. Additional funding was obtained from the Oregon Health Authority (OHA), a state government agency that oversees several priorities including substance use treatment, public health and Oregon’s delivery of Medicaid services, allowing for national recruitment for six additional cohorts in 2021 and 2022.

## Limitations

The small population size of this project limits the generalizability of the results and limits our ability to analyze the effects of confounders and differences in participant characteristics on overall outcomes. Also, since the recruitment was done through mass e-mails through various list serves and with overlap of members in the lists, we were not able to calculate the true response rate. Only a small self-selected group of dentists registered for this program, who must have had a particular interest in the topic beforehand. There was also an attrition issue with more attendees participating in the first sessions and gradually decreasing towards session 6. For the planning of future programs, we will capture additional qualitative data from participants to consider modifying content or session structure to reduce attrition. Further, sustainability of Project ECHO relies heavily on grant funding. Additionally, there was no follow-up with the participants to measure the program’s short- and long-term effects on sustained clinical behavior change for appropriate prescribing for pain management. Future ECHO programs could solicit data on clinical behavior change 6 months and 1 year after completion of the program. While the assumption is that a change in provider knowledge and behavior will result in greater patient satisfaction in pain management and provider-patient interaction, there was no patient participation in this study that allowed the measurement of patient-perceived outcomes as a result of changes in provider knowledge, attitudes and practices.

## Conclusions

The Pain Management and Substance Use Disorders Dental ECHO program was effective in rapidly disseminating evidence-based information. Dentists viewed this program as having a high degree of benefit for the optimal management of dental pain and the recognition of patients with substance use disorders. The perceived high degree of benefit, increased confidence, and positive clinical behavior change may result in more responsible opioid prescribing and dental pain management. Future ECHO programs intend to expand the scope and the reach by offering the program nationwide and update course content to remain applicable to participants across different states.

## Data Availability

The datasets used and/or analyzed during the study are available from the corresponding author on reasonable request.
